# Designing secure PUF-based authentication protocols for constrained environments

**DOI:** 10.1038/s41598-023-48464-z

**Published:** 2023-12-07

**Authors:** Sang-Woong Lee, Masoumeh Safkhani, Quynh Le, Omed Hassan Ahmed, Mehdi Hosseinzadeh, Amir Masoud Rahmani, Nasour Bagheri

**Affiliations:** 1 Pattern Recognition and Machine Learning Lab, Gachon University, 1342 Seongnamdaero, Sujeonggu, Seongnam, 13120 Republic of Korea; 2 Department of Computer Engineering, Shahid Rajaee Teacher Training University, Tehran, 16788-15811 Iran; 3 School of Computer Science, Institute for Research in Fundamental Sciences (IPM), P. O. Box 19395-5746 Tehran, Iran; 4 Institute of Research and Development, Duy Tan University, Da Nang, Vietnam; 5 Department of Information Technology, University of Human Development, Sulaymaniyah, Iraq; 6 School of Medicine and Pharmacy, Duy Tan University, Da Nang, Viet Nam; 7 Future Technology Research Center, National Yunlin University of Science and Technology, Yunlin, Taiwan; 8 Department of Electrical Engineering, Shahid Rajaee Teacher Training University, 16788-15811 Tehran, Iran

**Keywords:** Energy science and technology, Mathematics and computing

## Abstract

Physical Unclonable Functions (PUFs) are widely used in cryptographic authentication and key-agreement protocols due to their unique physical properties. This article presents a comprehensive cryptanalysis of two recently developed authentication protocols, namely PLAKE and EV-PUF, both relying on PUFs. Our analysis reveals significant vulnerabilities in these protocols, including susceptibility to impersonation and key leakage attacks, which pose serious threats to the security of the underlying systems. In the case of PLAKE, we propose an attack that can extract the shared secret key with negligible complexity by eavesdropping on consecutive protocol sessions. Similarly, we demonstrate an efficient attack against EV-PUF that enables the determination of the shared key between specific entities. Furthermore, we highlight the potential for a single compromised client in the EV-PUF protocol to compromise the security of the entire network, leaving it vulnerable to pandemic attacks. These findings underscore the critical importance of careful design and rigorous evaluation when developing PUF-based authentication protocols. To address the identified vulnerabilities, we present an improved PUF-based authentication protocol that ensures robust security against all the attacks described in the context of PLAKE and EV-PUF. Through this research, we contribute to the field by exposing vulnerabilities in existing PUF-based authentication protocols and offering an improved protocol that enhances security and safeguards against various attack vectors. This work serves as a valuable reference for researchers and practitioners involved in the design and implementation of secure authentication schemes for IoT systems and dynamic charging systems for electric vehicles.

## Introduction

The Internet of Things (IoT) has profoundly transformed our interaction with the environment, encompassing domains such as smart homes and industrial automation. Nevertheless, the widespread adoption of IoT devices has introduced novel security challenges, necessitating the development of lightweight and secure authentication protocols^1^. Conventional cryptographic solutions tend to be excessively resource-intensive and costly for IoT devices, which typically operate under resource constraints. Consequently, there is an increasing demand for alternative solutions that offer robust security^2^, while simultaneously being lightweight and cost-effective^3^.

Secure communication systems require authentication as a crucial component to ensure that only authorized users or processes can access sensitive information or resources. Authentication protocols typically involve the exchange of credentials, such as usernames and passwords, to verify the identity of the user or process^4^. However, recent research has exposed vulnerabilities in many authentication protocols that attackers can exploit by exploiting weaknesses in the protocol design or implementation^5,6^. Conducting such security analyses aims to contribute to the broader understanding and knowledge of the design of secure protocols. It is important to subject any security mechanism to thorough independent analysis before placing trust in its effectiveness, for instance.

PUFs have emerged as a promising solution for enhancing the security of IoT devices^7–9^. PUFs are hardware-based primitives that generate a unique and unpredictable response to a challenge, which can serve as a secret key for authentication purposes. In comparison to traditional cryptographic solutions, PUFs offer lightweight, cost-effective, and IoT device-compatible features. Consequently, PUFs have garnered significant attention for the design of cryptographic authentication and key agreement protocols due to their distinctive physical properties, which make them highly resistant to tampering and cloning attacks^10–15^. These protocols find wide application in various domains, including Internet of Things (IoT) systems^15^ and electric vehicle (EV) charging stations^16^. However, the security of PUF-based protocols relies heavily on the design and implementation of said protocols.

In this paper, we conduct a cryptanalysis of two recent PUF-based authentication protocols: PLAKE^15^ and EV-PUF^16^. PLAKE is a mutual authentication protocol specifically designed for IoT systems, while EV-PUF is an authentication protocol tailored for dynamic charging systems of electric vehicles. Our analysis exposes vulnerabilities in both protocols, including susceptibility to impersonation attacks and key leakage attacks, which could compromise the security of the systems they aim to safeguard. These findings emphasize the significance of meticulous design and thorough evaluation of PUF-based authentication protocols to ensure their security. Furthermore, we present an improved protocol for enhancing the security of such applications.

## Motivation

It is widely acknowledged that before any new security mechanism can be deemed trustworthy, its security must be thoroughly vetted by third-party experts to ensure it is not vulnerable to potential shortcomings. Two recently proposed authentication protocols, namely PLAKE^15^ and EV-PUF^16^, are notable for their reliance on physical unclonable functions (PUFs) as a foundational building block to provide robust security against a wide range of attacks, including cloning attacks. Additionally, both protocols leverage one-way hash functions to ensure data integrity. The designers of these schemes have claimed a high level of security, yet no independent security evaluations have been conducted to validate their assertions to the best of our knowledge. Given these shared characteristics, we are motivated to shed light on the security level of these protocols and determine whether they have indeed achieved their claimed level of security.

### Our contribution

The primary contribution of this paper is a comprehensive security evaluation of two recently proposed authentication protocols that rely on PUFs, namely PLKE and EV-PUF.

Our analysis of PLKE reveals a novel attack that enables efficient recovery of secret parameters for this protocol. Specifically, our proposed attack allows an adversary to determine the shared key for all sessions after a minimum of two consecutive sessions, based solely on the messages transmitted over the public channel. The attack is passive and has negligible complexity, and can also be extended to enable impersonation and desynchronization attacks.

In addition, our security evaluation of EV-PUF exposes several vulnerabilities that undermine the security guarantees claimed by the protocol. Specifically, we demonstrate that the protocol does not provide forward secrecy and is susceptible to attacks by privileged insider adversaries, impersonation attacks, and session key compromise. Furthermore, we show that compromising a single node within the network can have a significant impact on the security of all other nodes, rendering the protocol vulnerable to pandemic attacks^17^. All of the proposed attacks are highly efficient and easy to execute.

The paper presents a novel PUF-based authentication protocol designed to provide secure authentication and key-agreement for IoT systems or electric vehicle charging systems. The protocol leverages the unique physical properties of PUFs combined with a sound but lightweight cipher suite (Ascon^18^) to establish mutual authentication and generate shared secret keys.

### Paper orgnaization

The remainder of this paper is structured as follows. Section “Preliminaries” provides an overview of PUFs and their applications in Internet of Things (IoT) security, as well as a description of target protocols. In this section, we also introduce adversary models. In Sections “Security analysis of PLAKE” and “Security analysis of EV-PUF”, we discuss, respectively, the security analysis of the PLAKE and EV-PUF protocols. In Sections “PUF-Based Mutual Authentication Protocol”, taking the lessons learned from our analysis, we propose a new PUF-based mutual authentication protocol and its security and cost analysis are given in Sections “PUF-Based Mutual Authentication Protocol”. Finally, we conclude the paper in Section “Conclusion”.

## Preliminaries

In this section, the required preliminaries in this paper are briefly reviewed. Table 1 represents the notations used in this paper.

**Table 1:** List of used notations.

Symbol	Description
$\mathscr {C}_i$	*i* *th* client
U, EV, TSP, RSU, RCS, CP	Respectively denotes Users (drivers), Electric Vehicle, Trusted Service Provider, Region Charging Server, Road Side Units and Charging Pads
$ID_{x}$	Identity of the target entity *x*
*PW*	The user password
$SK_{x}$	Long term secret key of the target entity *x*
$PUF^x(\cdot )$	Evaluation of the embedded PUF function on entity *x*
$(C_x,R_x)$	Challenge and the PUF response (CRP), i.e. $R_x=PUF^x(C_x)$
$\mathscr {H}(\cdot )$	One-way hash function
$(PU_x,PR_x)$	Public and private keys of the target entity *x*
$KDF(\cdot )$	Key derivation function
*sk*	The session key
$\tau$	A threshold value for Hamming distance
$FHD(\cdot )$	Hamming distance function
*T*	Timestamp
$\mathscr {C}$	A client, e.g. an IoT device
$\oplus$	XOR operation
$\Vert$	Concatenation

### An overview on PUF

A Physical Unclonable Function (PUF) is a hardware security primitive that exploits the unique and unpredictable variations in physical characteristics of a device to generate a unique identifier or a cryptographic key. PUFs can be classified based on different criteria, such as their operating principle, the type of physical variations they exploit, and the type of output they produce^19, 20^.

One classification is based on the operating principle, where PUFs can be divided into two main categories: challenge-response PUFs and true random number generators (TRNGs) with PUF-based entropy sources. Challenge-response PUFs generate a response to a challenge input based on the unique physical characteristics of the device, while TRNGs with PUF-based entropy sources extract randomness from the physical variations in the device.

Another classification is based on the type of physical variation exploited by PUFs. PUFs can exploit various physical characteristics, such as delay, noise, power consumption, and ring oscillator frequencies, among others. For example, a ring oscillator (RO) PUF exploits the variations in the oscillation frequency of a ring oscillator circuit due to manufacturing process variations or environmental factors.

PUFs can also be classified based on the type of output they produce. PUFs can produce binary or multi-bit responses, depending on the application requirements. For example, a binary PUF produces a single-bit response, while a multi-bit PUF produces multiple bits of output.

Some commonly used PUFs include the Arbiter PUF, the RO PUF, and the Magnetic PUF, among others. The performance and security characteristics of PUFs depend on various factors, such as design parameters, the quality of the physical variations exploited, and environmental conditions.

PUFs have emerged as a promising solution for improving the security of devices in the Internet of Things (IoT) due to their low power consumption, small footprint, and resistance to physical and side-channel attacks^21–23^.

PUFs have found numerous applications in IoT security, including secure bootstrapping, secure firmware updates, secure key exchange, and secure communication. For example, PUFs can be used to generate unique device identities that are resistant to cloning and counterfeiting, allowing secure device authentication and access control^23^. PUF-based key exchange protocols can be used to establish secure communication channels between IoT devices^24–28^. Furthermore, recent research has focused on developing new PUF-based security mechanisms that address the limitations of traditional PUFs. For example, PUFs based on machine learning have been proposed that are more resistant to modeling attacks^29^. Recent surveys and reviews have provided a comprehensive analysis of the state of the art of PUFs, including their architectures, protocols, and security for Internet of Things (IoT) applications^23,30^. These publications have investigated the role of PUFs in IoT security, analyzed PUF-based threats on IoT devices, discussed possible defense strategies, and presented existing PUF architectures and authentication protocols using PUFs. They have also evaluated the progress, challenges, and future expectations of PUF-based security protocols for IoT devices^23,30^.

In summary, PUFs are a promising security primitive for IoT devices that can be used to enhance the security of authentication, key exchange, and data protection protocols. Recent research has shown increasing interest in PUF-based security mechanisms, and further exploration of their potential in IoT security is ongoing.

### Adversary model

The security of the protocols is evaluated using two different adversary models: the adversary model “Dolev–Yao (DY)”^31^ and the “Canetti-Krawczyk (CK)”^32^ adversary model.

In the DY adversary model, the adversary is assumed to have complete control over the communication channels between parties but does not have access to their internal values. This means that the adversary can intercept, modify, or replay messages between the parties but cannot extract any secret information from them.

In contrast, the CK adversary model is a stronger adversary model that allows the adversary to extract secret credentials and compromise established session keys. This means that the adversary can obtain access to the internal values of the parties and use this information to compromise the security of the protocol.

By evaluating the security of the protocols under both adversary models, we can ensure whether they provide sufficient protection against attacks from both weaker and stronger adversaries.

Another attack model which is considered in this paper is the pandemic session key disclosure attack. Bagheri * et al.* introduced the concept of a pandemic attack^17^, which includes the pandemic session key disclosure attack. This attack occurs when an adversary has compromised a client $\mathscr {C}_i\in \mathscr {C}$ and is attempting to establish a session key with a different client $\mathscr {C}_j\ne \mathscr {C}_i$.

### PLAKE

Because of the rising amount of cyber assaults, the security of Internet of Things (IoT) systems has become a serious problem. Before transmitting sensitive data, mutual authentication confirms the identity of both the device and the server. Physical unclonable functions (PUFs) have emerged as a potential alternative to the mutual authentication of an IoT system. PUFs are hardware-based security primitives that provide unique and unpredictable answers to challenge input, allowing devices and services to be authenticated^33^. In this section, we briefly review the PLAKE^15^ which is a PUF-based mutual authentication protocol for IoT systems. The PLAKE protocol runs in two phases: a one-time enrollment phase and a device authentication and key exchange phase.

#### One-Time Enrollment (OTE) phase

This phase which is accomplished in a secure channel and only once time before IoT devices deployment runs as below:

1. The server generates a PUF challenge $C^i_A$ for $\mathscr {C}_A$ for instance;
2. retrieves the PUF response i.e. $R^i_A=PUF^{\mathscr {C}}(C^i_A)$;
3. and saves the PUF-CRP i.e. the couple $(C^i_A,R^i_A)$ in the secure memory of IoT Device ($ID_{<\mathscr {C}_A>}$).

####  Device Authentication and Key Exchange Phase

This phase compromises two steps: node-to-node (N2N) communication and node-to-server (N2S) communication.

**Node to Node (N2N) Communication:**

1. Connection Initialization This step runs as below:
  The communicator IoT device ($\mathscr {C}_A$) sends a connection request message $<ID_A>$ to $\mathscr {C}_B$;
  Once received the message, $\mathscr {C}_B$ sends N2N connection request $<ID_A,ID_B>$ to the server.
  The server extracts the corresponding PUF CRPs of $\mathscr {C}_A$ and $\mathscr {C}_B$ from its memory, e.g. $(C^i_{A},R^i_{A})$ and $(C^i_{B},R^i_{B})$.
  The sever produces two random values as $RN^i$ and $sk^i_{AB}$ and then calculates $M^i_A=R^i_{A}\oplus RN^i$, $M^i_B=R^i_{B}\oplus RN^i$, $Y^i_{AB}=sk^i_{AB}\oplus RN^i$, $H^i_A=\mathscr {H}(R^i_{A}\Vert M^i_A)$ and $H^i_B=\mathscr {H}(R^i_{B}\Vert M^i_B)$.
  The server sends $<C^i_{A}, M^i_A,M^i_B,H^i_A,H^i_B, Y^i_{AB}>$ to $\mathscr {C}_A$ and $<C^i_{B}, M^i_A,M^i_B,H^i_A,H^i_B, Y^i_{AB}>$ to $\mathscr {C}_B$.
2. Server authentication In these steps $\mathscr {C}_A$ and $\mathscr {C}_B$ authenticate the server through following steps:
  $$\mathscr {C}_{A}$$ recomputes $$H'_A$$ and $$H'_B$$;
  Checks whether $H'_A{\mathop {=}\limits ^{?}}H^i_A$ and $H'_B{\mathop {=}\limits ^{?}}H^i_B$.
  If they are equal, $\mathscr {C}_A$ successfully authenticates the server.
  $\mathscr {C}_B$ also authenticates the server, as mentioned about $\mathscr {C}_A$.
3. $\mathscr {C}_A$ and $\mathscr {C}_B$ Mutual Authentication
  Once the server authentication completed, $\mathscr {C}_A$ and $\mathscr {C}_B$ extract $sk^i_{AB}$ from the recived messages.
  $\mathscr {C}_A$ generates $Y^i_B=sk^i_{AB}\oplus R^i_{B}$ and sends it to $\mathscr {C}_B$.
  Once the message has been received, $\mathscr {C}_B$ extracts $R^i_{B}$ as $R^i_{B}=sk^i_{AB}\oplus Y^i_B$ and then checks whether it is equal to its PUF-generated response to the challenge $C^i_{B}$ given by the server, to successfully authenticate $\mathscr {C}_A$. Then $\mathscr {C}_B$ generates $Y^i_A=sk^i_{AB}\oplus R^i_{A}$ and sends it to $\mathscr {C}_A$.
  Once the message has been received, $\mathscr {C}_A$ extracts $R^i_{A}$ as $R^i_{A}=sk^i_{AB}\oplus Y^i_A$ and then checks whether it is equal to its PUF-generated response to challenge $C^i_{A}$ given by the server, to authenticate $\mathscr {C}_B$.
  Once the nodes $\mathscr {C}_A$ authenticated the server and also mutually authenticate $\mathscr {C}_B$, it goes to the update phase to update its CRP responses to $C^{i+1}_{A}=C^i_{A}\oplus R^i_{A}$ and $R^{i+1}_{A}=PUF^A(C^{i+1}_{A})$. Then $\mathscr {C}_A$ calculates $M^i_{SA}= R^{i+1}_{A} \oplus RN^i$ and $H^i_{SA}=\mathscr {H}(R^{i+1}_{A}\Vert M^i_{SA})$, and sends $<M^i_{SA}, H^i_{SA}>$ to the server. Similarly, $\mathscr {C}_B$ authenticates the server and $\mathscr {C}_A$ and updates its CRP responses to $C^{i+1}_{B}=C^i_{B}\oplus R^i_{B}$ and $R^{i+1}_{B}=PUF^B(C^{i+1}_{B})$, calculates $M^i_{SB}= R^{i+1}_{B} \oplus RN^i$ and $H^i_{SB}=\mathscr {H}(R^{i+1}_{B}\Vert M^i_{SB})$, and sends $<M^i_{SB}, H^i_{SB}>$ to the server.
4. Server authentication of $\mathscr {C}_A$ and $\mathscr {C}_B$ The server authenticates both IoT devices and also updates the secure PUF CRP database as follows:
  - Upon receiving the messages, the server uses the bit sequences $M_{SA}$ and $M_{SB}$ to generate updated PUF responses from $\mathscr {C}_A$ and $\mathscr {C}_B$ as $R^{i+1}_A= RN^i \oplus M_{SA}$ and $R^{i+1}_B= RN^i \oplus M_{SB}$;
  - Computes $H'_{SA}$ and $H'_{SB}$ and checks whether $H'_{SA}{\mathop {=}\limits ^{?}}H_{SA}$ and $H'_{SB}{\mathop {=}\limits ^{?}}H_{SB}$; After verifying the hash values, the server successfully authenticates both $\mathscr {C}_A$ and $\mathscr {C}_B$.
  - The server then updates the stored challenges $C^i_A$ and $C^i_B$ to $C^{i+1}_A= C^i_A \oplus R^i_A$ and $C^{i+1}_B=C^i_B \oplus R^i_B$, respectively.
  - The server replaces and saves $<ID_A,C^{i+1}_A, R^{i+1}_A>$ $and <ID_B,C^{i+1}_B, R^{i+1}_B>$ .
5. Set session key After successful authentication between communicating nodes, $sk^i_{AB}$ acts as a session key for secure communication between neighboring nodes. This private key is updated each time a new session is established.

**Node to Server (N2S) Communication:**

1. Connection Initialization IoT device $\mathscr {C}_B$ with the identifier of $ID_B$ sends a connection request to the server. After receiving the request, the server retrieves the stored PUF-CRP, i.e. $(C^i_B, R^i_B)$ using $ID_B$ and then generates a random number *RN* and computes $MB=R^i_B \oplus RN$ and $H_B= \mathscr {H}(R^i_B\Vert M_B)$. Then the server sends $<C^i_B,M_B, H_B>$ to $\mathscr {C}_B$.
2. Server Authentication After receiving the message, $\mathscr {C}_B$ first forwards $C^i_B$ to its embedded PUF instance and generates the corresponding $R^i_B=PUF^B(C^i_B)$. $\mathscr {C}_B$ extracts *RN* as $R^i_B \oplus RN$ and then checks received $H_B$ to authenticate the server. If it is held, $\mathscr {C}_B$ will create a PUF-CRP and send it to the server. $\mathscr {C}_B$ computes $C^{i+1}_B= C^i_B \oplus R^i_B$ and receives its PUF response as $R^{i+1}_B=PUF^B(C^{i+1}_B)$. Then it calculates $M_{SB}=R^{i+1}_B\oplus RN$ and $H_{SB} =\mathscr {H}(R^{i+1}_B\Vert M_{SB})$ and sends $<M_{SB}, H_{SB}>$ to the server.
3. Node Authentication The server extracts $R^{i+1}_B$ as $RN\oplus M_{SB}$ and verifies $H_{SB}$. If it is correct, the server successfully authenticates $\mathscr {C}_B$ and updates the challenge $C^{i+1}_B=C^i_B \oplus R^i_B$ and $R^{i+1}_B=PUF^B(C^{i+1}_B)$. Finally, the server updates the secure PUF-CRP database.
4. Set session key After completing server authentication and node authentication in the N2S protocol, the server and IoT devices can establish secure communication using $R^{i+1}_B$ as the private key for this particular session which is updated with each new session.

### EV-PUF

The Intelligent Transportation System (ITS) facilitates the communication between vehicles through Vehicular to Vehicular (V2V) and Vehicular to Infrastructure networks^34^. Wireless Power Transfer (WPT) technology has emerged as a promising solution to charge electric vehicles (EV) in ITS, allowing electric vehicles to charge their batteries while driving. Inductive power transfer technology (IPT) is a type of WPT that has been shown to be effective for EV charging^35^. For example, Sweden is currently building the world’s first permanent electrified road for EVs that utilizes an inductive charging system buried under the road surface to send electricity to a coil in the EV^36^. Electrified roadways, which integrate wireless charging infrastructure into asphalt, have the potential to enable EVs to operate continuously with unlimited power.

The EV-PUF protocol is an authentication protocol designed for the dynamic charging systems of electric vehicles, which utilizes a lightweight approach based on PUF technology. The protocol involves five key components, namely the Trusted Service Provider (TSP), Region Charging Server (RCS), Road Side Units (RSUs), Electric Vehicle (EV), Charging Pads (CP), and users (drivers). The proposed protocol includes many steps but we just explain those parts that are required to understand the proposed attacks. An interested reader could find all details of the EV-PUF in Ref.^16^. It should be noted EV-PUF uses two variants of PUF, i.e. RPUF, and WPUF. However, it has no effect on our attack, and the proposed attacks work for any type of PUF. Hence, for the sake of simplicity of notation, we just use $PUF(\cdot )$ to describe both.

#### Initialization

In this phase of the protocol, the TSP selects a secure cryptographic hash function $\mathscr {H}(\cdot )$, private keys $<SK_{RSU_i},SK_{TCS_i},SK_{EV}>$. It also selects a group key $G_{pad}$ and forwards it to each RSU and each CP.

#### RSU registration

To register an RSU, the RSU generates a random identifier $ID_{RSU_i}$ and sends it to TSP. The TSP computes $K_{RSU_i}=\mathscr {H}(ID_{RSU_i}\Vert SK_{RSU_i})$ and stores $ID_{RSU_i}$ and $K_{RSU_i}$ in a table $T_{RSU}$. Then it generates $PU_{RSU_i}$ and $PR_{RSU_i}$ respectively as the public and private key of ${RSU_i}$ and stores them. Next, TSP sends $< PU_{RSU_i}, PR_{RSU_i}, K_{RSU_i},\mathscr {H}(SK_{RSU_i})>$ to RSU.

#### RCS registration

To register an RCS, it generates a random identifier $ID_{RCS_i}$ and sends it to TSP. The TSP computes $K_{RCS_i}=\mathscr {H}(ID_{RCS_i}\Vert SK_{RCS_i})$ and stores $ID_{RCS_i}$ and $K_{RCS_i}$ in $T_{RCS}$, as a table. Then generates $PU_{RCS_i}$ and $PR_{RCS_i}$, respectively, as the public and private key of ${RCS_i}$ and stores them. Finally, TSP, sends $< PU_{RCS_i}, PR_{RCS_i}, K_{RCS_i},\mathscr {H}(RCS_i)>$ to RCS.

#### User registration

During the user registration, the user generates a random password $PW_u$ and identifier $ID_u$ and forwards them to TSP. The TSP computes $i=\mathscr {H}(PW_u\Vert ID_u)$, selects a secret *P* and a random number *z*, computes $L=\mathscr {H}(i\Vert P)$ and $SID=\mathscr {H}(z)$ and stores $<ID_u,P,SID>$ in a smart-card *SC* and $<L,P,SID>$ in EV.

#### EV registration

To register an EV, it selects a random identifier $$ID_{EV}$$ and a random number $$R_{EV}$$ to compute $$PID_{EV}=\mathscr {H}(ID_{EV}\Vert R_{EV})$$ and $$Y_{EV}=R_{EV}\oplus \mathscr {H}(ID_{EV})$$ and send $$<PID_{EV}>$$ to TSP. The TSP computes $$Q_S=\mathscr {H}(PID_{EV}\Vert SK_{EV})$$ and $$C_{EV}=\mathscr {H}(PID_{EV}\Vert W_S)$$. Next, $$<ID_{RCS_i},ID_{RSU_i},R_S>$$ is stored in a table entitled $$T_{EV}$$ and $$<PID_{EV},C_{EV},T_{R=1}>$$ is stored in a table entitled $$T_{C}$$. Then, it produces two challenges $$C_i$$ and $$C_x$$ and sends $$<C_i,C_x, W_S,T_{EV}, \mathscr {H}(SK_{RSU_i})>$$ to EV. The EV computes $$X_s=W_S\oplus PID_{EV}$$ and $$C_{EV}=\mathscr {H}(PID_{EV}\Vert W_S)$$ and stores $$X_S$$ instead of $$W_S$$. Then, it selects a random key $$r_i$$ to compute $$R_x=PUF^{EV}(C_x)$$, $$k_i=\mathscr {H}(R_x\Vert r_i)$$, $$C=C_i\oplus k_i$$, $$R_i\oplus PUF(C)$$ and store $$<X_S,Y_{EV}, T_{EV}, C_{EV}, \mathscr {H}(SK_{RSU_i})>$$ in a tamper-proof memory and sends $$<PID_{EV}, R_i,K_i, R_x)>$$ to TSP. Once the message has been received, TSP stores $$<PID_{EV}, C_i, R_i,k_i, R_x>$$ in a table entitled $$T_{CRP}$$.

#### User authentication

In this phase of the protocol, the user / owner of the EV inserts $PW_u$ and given the timestamp $T_c$, SC computes $i=\mathscr {H}(PW_u\Vert ID_u)$, $L=\mathscr {H}(i\Vert P)$ and $K=\mathscr {H}(L\Vert T_c\Vert SID)$ and sends $<K\Vert T_c\Vert SID>$ to EV. EV verifies whether $T_c-T_r<T$ and also the received *K* to authenticate the user and update *SID* as $SID^{new}=\mathscr {H}(SID\Vert P)$ in its database and in the SC’s memory.

#### Login and mutual authentication

This phase of the protocol takes place between EV and RSU. On the EV side, the identifier $ID_{EV}$ is inserted to compute $R_{EV}=Y_{EV}\oplus \mathscr {H}(ID_{EV})$, $PID_{EV}=\mathscr {H}(ID_{EV}\Vert R_{EV})$ and $W_S=X_S\oplus PID_{EV}$ and verify whether $C_{EV}=\mathscr {H}(PID_{EV}\Vert W_S)$. Next, $ID_{RSU_i}$ and $R_S$ are taken from $T_{EV}$ to extract $Q_S=R_S\oplus PID_{EV}$. Then, two random values $RN_1$ and $RN_2$ are generated to calculate $B=PID_{EV}\oplus \mathscr {H}(ID_{RSU_i}\Vert n-1\Vert \mathscr {H}(SK_{RSU_i}))$, $D=\mathscr {H}(PID_{EV}\Vert Q_S\Vert RN_1\Vert B)$, $R_x=PUF(C_x)$, $n^*_2=RN_2\oplus k_i$, and $V_1=\mathscr {H}(n^*_2\Vert k_i)$. Then it sends $<B,D,RN_1,n^*_2,V_1>$ toward RSU.

Once the message has been received, the RSU computes $PID_{EV}=B\oplus \mathscr {H}(ID_{RSU_i}\Vert n-1\Vert \mathscr {H}(SK_{RSU_i}))$, $Q_S=\mathscr {H}(PID_{EV}\Vert K_{RSU_i})$ and verifies whether $D=\mathscr {H}(PID_{EV}\Vert Q_S\Vert RN_1\Vert B)$ accepts the request and achieves the parameters $<C_i,R_i,k_i,R_x>$ from the TSP, verifying $V_1$, choosing a random number $RN_3$, computing $RN_2=n^*_2\oplus k_i$, $n^*_3=RN_3\oplus k_i$, $R^{1*}_i=R^1_i\oplus k_i$ and $V_2=\mathscr {H}(n^*_3\Vert k_i\Vert R^{1*}_i\Vert RN_2)$. Then the RSU sends $<C_i,R^{1*}_i,n^{*}_3,V_2>$ to EV.

EV computes $RN_3=n^*_3\oplus k_i$ and $R^1_i=R^{1*}_i\oplus k_i$ to verify $V_2$ and authenticate RSU. Next, it computes $C=C_i\oplus k_i$, $R^{1+}_i\Vert R^{2+}_i=PUF(C)$ and verifies whether $FHD(R^{1+}_i,R^{1}_i)<\tau$. Next, $X=R^{2+}_i\oplus k_i$, $C_{i+1}=\mathscr {H}(C\Vert RN_2\Vert RN_3)$, $R_{i+1}=PUF(C_{i+1})$, $R^{*}_{i+1}=R_{i+1}\oplus k_i$, and $V_3=\mathscr {H}(k_i\Vert R^{*}_{i+1}\Vert RN_3\Vert X)$ are calculated and $<R^{*}_{i+1},X,V_3>$ is sent to RSU. The session key is defined as $SK=\mathscr {H}(RN_2\Vert RN_3\Vert R^1_i\Vert R^{2+}_i)$.

RSU verifies the received $V_3$, computes $R^{2+}_{i}=X\oplus k_i$ and verifies whether $FHD(R^{2+}_{i},R^{}_{i})<\tau$. Then, $C=C_i\oplus k_i$, $C_{i+1}=\mathscr {H}(C\Vert RN_2\Vert RN_3)$ and $R_{i+1}=R^{*}_{i+1}\oplus k_i$ are computed and the session key is driven as $SK=\mathscr {H}(RN_2\Vert RN_3\Vert R^1_i\Vert R^{2+}_i)$.

After mutual authentication with RSU, the authenticated EV can request a charge by sending $<CH^i_{req},N_{ct}>$. In response, RSU chooses a seed $S_i$ to compute $KDF_{SK}(S_i)=K_1\Vert K_2$ and $V_4=\mathscr {H}(K_1\Vert K_2\Vert N_ct)$ and send $<S_i,V_4>$ to EV. It also computes $Tag=\mathscr {H}(K_1\Vert K_2\Vert PID_{EV}\Vert ID_{RSU_i}\Vert N_{ct})$ and sends $<E_{Gpad}(Tag)>$ to the $1^{st}$ CP. Once received the message, EV once again computes $KDF_{SK}(S_i)=K_1\Vert K_2$ and $V_4=\mathscr {H}(K_1\Vert K_2\Vert N_ct)$ to verify the correctness of $V_4$. Then it calculates $Tag=\mathscr {H}(K_1\Vert K_2\Vert PID_{EV}\Vert ID_{RSU_i}\Vert N_{ct})$ and sends $<Tag>$ to the $1^{st}$ CP. The first CP compares the *Tag* received from EV and the encrypted one from RSU.

#### Handover

The handover phase occurs if the EV moves from an RSU to another RSU or from an RCS to another RCS. In this regard, and when the EV moves from $RSU_i$ to $RSU_j$, the EV sends a handover request to $RSU_i$, where it finds the nearest RSU, for example, $RSU_j$, and sends $<RN_4,ID_{RSU_j}>$ to the EV and sends $<E_{PU_{RSU_j}}(PIDV_{EV},RN_4,ID_{RSU_i})>$ to $RSU_j$. Using its $T_{EV}$, the EV cross-checks the received $ID_{RSU_j}$ to compute $G=\mathscr {H}(PID_{EV}\Vert ID_{RSU_i}\Vert RN_4\Vert ID_{RSU_j})$ and send it to $RSU_j$ and also compute $SK_{T}=\mathscr {H}(PID_{EV}\Vert RN_4)$. On the other hand, $RSU_j$ decrypts the received message from $RSU_i$ and once again computes $G=\mathscr {H}(PID_{EV}\Vert ID_{RSU_i}\Vert RN_4\Vert ID_{RSU_j})$ to authenticate EV and compute $SK_{T}=\mathscr {H}(PID_{EV}\Vert RN_4)$.

In a similar approach, when the EV moves from $RCS_i$ to $RCS_j$, EV sends a handover request to $RCS_i$, where it finds the nearest RCS, e.g. $RCS_j$, and generates $RN_5$ and computes $V_5=\mathscr {H}(RN_5\Vert K_{RCS_j}\Vert ID_{RCS_j})$ and sends $<ID_{RCS_j},V_5>$ to EV. It also sends $<E_{PU_{RCS_j}}(PID_{EV},RN_5,ID_{RCS_i},V_5)>$ to $RCS_j$. Using its $T_{EV}$, the EV crosses check the received $ID_{RCS_j}$ to compute $L=\mathscr {H}(PID_{EV}\Vert ID_{RCS_i}\Vert V_5\Vert ID_{RCS_j})$ and sends it to $RCS_j$. Once received the messages, $RCS_j$ decrypts the received message from $RCS_i$ and once again computes $L=\mathscr {H}(PID_{EV}\Vert ID_{RCS_i}\Vert V_5\Vert ID_{RCS_j})$ to authenticate EV.

## Security analysis of PLAKE

In^15, TABLE IV]^, the designers claimed the security of PLAKE against various attacks, including impersonation, replay, secret key extraction, denial of service and PUF-CRP extraction. In this section, we evaluate the security of PLAKE in an adversary model similar to that of the designers^15, Sec. III.B]^, i.e. YD and CK models to shed light on the protocol security from an independent third-party point of view.

### Secret disclosure attack

In the authentication phase described in Section  “PLAKE”, the server generates two nonces, denoted $RN^i$ and $sk^i_{AB}$. The server then computes several values, including $M^i_A=R^i_{A}\oplus RN^i$, $M^i_B=R^i_{B}\oplus RN^i$, $Y^i_{AB}=sk^i_{AB}\oplus RN^i$, $H^i_A=\mathscr {H}(R^i_{A}\Vert M^i_A)$, and $H^i_B=\mathscr {H}(R^i_{B}\Vert M^i_B)$. The server sends the message $<C^i_{A}, M^i_A,M^i_B,H^i_A,H^i_B, Y^i_{AB}>$ to $\mathscr {C}_A$ and the message $<C^i_{B}, M^i_A,M^i_B,H^i_A,H^i_B, Y^i_{AB}>$ to $\mathscr {C}_B$ through a public channel. These values are used by the nodes to authenticate the server.

Once $\mathscr {C}_A$ and $\mathscr {C}_B$ have authenticated the server and each other, they proceed to the update phase, where they update their CRP responses. Specifically, $\mathscr {C}_A$ updates its CRP response to $C^{i+1}_{A}$ as $C^i_{A}\oplus R^i_{A}$ and its $R^{i+1}_{A}$ as $PUF^A(C^{i+1}_{A})$; similarly $\mathscr {C}_B$ updates its CRP response to $C^{i+1}_{B}$ as $C^i_{B}\oplus R^i_{B}$ and its $R^{i+1}_{B}$ as $PUF^B(C^{i+1}_{B})$. Then, $\mathscr {C}_A$ and $\mathscr {C}_B$ respectively calculate $M^i_{SA}= R^{i+1}_{A} \oplus RN^i$, $H^i_{SA}=\mathscr {H}(R^{i+1}_{A}\Vert M^i_{SA})$ and $M^i_{SB}= R^{i+1}_{B} \oplus RN^i$, and $H^i_{SB}=\mathscr {H}(R^{i+1}_{B}\Vert M^i_{SB})$, and respectively sends $<M^i_{SA},H^i_{SA}>$ and $<M^i_{SB},H^i_{SB}>$ to the server.

Now suppose that an adversary has eavesdropped on two consecutive sessions between $\mathscr {C}_A$, $\mathscr {C}_B$, and the server, namely the *i* *th* and $(i+1){th}$ sessions. In this case, the adversary has access to the information that was transmitted over the public channel during the *i* *th* session.

$$\begin{aligned} C^i_{A}{} & {} \\ C^i_{B}{} & {} \\ M^i_A = &\; {} R^i_{A}\oplus RN^i\\ M^i_B = &\; {} R^i_{B}\oplus RN^i\\ Y^i_{AB} = &\; {} sk^i_{AB}\oplus RN^i \\ H^i_A = &\; {} \mathscr {H}(R^i_{A}\Vert M^i_A)\\ H^i_B = &\; {} \mathscr {H}(R^i_{B}\Vert M^i_B)\\ Y^i_B = &\; {} sk^i_{AB}\oplus R^i_{B}\\ Y^i_A = &\; {} sk^i_{AB}\oplus R^i_{A}\\ M^i_{SA} = &\; {} R^{i+1}_{A} \oplus RN^i\\ M^i_{SB} = &\; {} R^{i+1}_{B} \oplus RN^i\\ H^i_{SA} = &\; {} \mathscr {H}(R^{i+1}_{A}\Vert M^i_{SB}) \\ H^i_{SB} = &\; {} \mathscr {H}(R^{i+1}_{B}\Vert M^i_{SB}) \end{aligned}$$

where,

$$\begin{aligned} C^{i+1}_{A} = &\; {} C^i_{A}\oplus R^i_{A}\\ C^{i+1}_{B} = &\; {} C^i_{B}\oplus R^i_{B}\\ R^{i+1}_{A} = &\; {} PUF^A(C^{i+1}_{A})\\ R^{i+1}_{B} = &\; {} PUF^B(C^{i+1}_{B})\\ \end{aligned}$$

It is important to note that the information that was transferred over the public channel during the $(i+1){th}$ session may not be accessible to the adversary, as it was transmitted after the eavesdropping occurred. However, assuming that the adversary has access to the information that was transmitted during the $(i+1){th}$ session because the attack was passive which increases the adversary’s chance, then it has

$$\begin{aligned} C^{i+1}_{A}{} & {} \\ C^{i+1}_{B}{} & {} \\ M^{i+1}_A = &\; {} R^{i+1}_{A}\oplus RN^{i+1}\\ M^{i+1}_B = &\; {} R^{i+1}_{B}\oplus RN^{i+1}\\ T'^{i+1}_{AB} = &\; {} sk^{i+1}_{AB}\oplus RN^{i+1} \\ H^{i+1}_A = &\; {} \mathscr {H}(R^{i+1}_{A}\Vert M^{i+1}_A)\\ H^{i+1}_B = &\; {} \mathscr {H}(R^{i+1}_{B}\Vert M^{i+1}_B)\\ Y^{i+1}_B = &\; {} sk^{i+1}_{AB}\oplus R^{i+1}_{B}\\ Y^{i+1}_A = &\; {} sk^{i+1}_{AB}\oplus R^{i+1}_{A}\\ M^{i+1}_{SA} = &\; {} R^{i+2}_{A} \oplus RN^{i+1}\\ M^{i+1}_{SB} = &\; {} R^{i+2}_{B} \oplus RN^{i+1}\\ H^{i+1}_{SA} = &\; {} \mathscr {H}(R^{i+2}_{A}\Vert M^{i+1}_{SB}) \\ H^{i+1}_{SB} = &\; {} \mathscr {H}(R^{i+2}_{B}\Vert M^{i+1}_{SB}) \end{aligned}$$

where,

$$\begin{aligned} R^{i+2}_{A} = &\; {} PUF^A(C^{i+2}_{A})\\ R^{i+2}_{B} = &\; {} PUF^B(C^{i+2}_{B})\\ C^{i+2}_{A} = &\; {} C^{i+1}_{A}\oplus R^{i+1}_{A}\\ C^{i+2}_{B} = &\; {} C^{i+1}_{B}\oplus R^{i+1}_{B}\\ \end{aligned}$$

Given those data, the adversary is able to retrieve the following information:

$$\begin{aligned} R^i_{A} = &\; {} C^i_{A}\oplus C^{i+1}_{A}\\ RN^i = &\; {} R^i_{A}\oplus M^i_A\\ R^i_{B} = &\; {} M^i_B \oplus RN^i\\ sk^i_{AB} = &\; {} Y^i_{AB}\oplus RN^i \\ R^{i+1}_{A} = &\; {} M^i_{SA} \oplus RN^i\\ R^{i+1}_{B} = &\; {} M^i_{SB} \oplus RN^i\\ \end{aligned}$$

As mentioned earlier, $sk^i_{AB}$ is the session key that was used during the *i* *th* session, and the adversary has access to $R^{i+1}_{A}$ and $R^{i+1}_{B}$ also. With this information, the adversary could potentially compromise the $(i+1){th}$ session, as follows:

$$\begin{aligned} C^{i+1}_{A}{} & {} \\ RN^{i+1} = &\; {} R^{i+1}_{A}\oplus M^{i+1}_A\\ sk^{i+1}_{AB} = &\; {} T'^{i+1}_{AB}\oplus RN^{i+1} \\ R^{i+2}_{A} = &\; {} M^{i+1}_{SA}\oplus RN^{i+1}\\ R^{i+2}_{B} = &\; {} M^{i+1}_{SB} \oplus RN^{i+1}\\ C^{i+2}_{A} = &\; {} C^{i+1}_{A}\oplus R^{i+1}_{A}\\ C^{i+2}_{B} = &\; {} C^{i+1}_{B}\oplus R^{i+1}_{B}\\ \end{aligned}$$

which $sk^{i+1}_{AB}$ is the session key that has been used during the $i+1{th}$ session. In fact, if the adversary is able to compromise the $(i+1){th}$ session using the techniques described above, then it may be able to use the information obtained from that session to compromise subsequent sessions as well, leading to complete retrieval of the secret parameters or data that are transferred between $\mathscr {C}_A$, $\mathscr {C}_B$, and the server.

This passive attack is particularly dangerous because it is hard to detect, and the adversary is able to retrieve sensitive information without actively participating in the communication process. To prevent such attacks, it is crucial to use strong encryption techniques to protect the communication channels and authenticate all parties involved using secure and robust methods. Additionally, it may be helpful to periodically update the secret parameters and keys used in the protocol to further enhance the security of the system.

### Impersonation attack

If the adversary has access to the secret parameters $\{R^{i+2}_{A};R^{i+2}_{B};C^{i+2}_{A};C^{i+2}_{B}\}$, it could potentially impersonate the server, $\mathscr {C}_A$, and $\mathscr {C}_B$ in future sessions.

With this information, the adversary could compute the updated CRP responses for $\mathscr {C}_A$ and $\mathscr {C}_B$ in the $(i+2){th}$ session and impersonate the server to manipulate these responses. This would allow the adversary to gain access to sensitive information or compromise the integrity of the system.

### Desynchronization

To desynchronize $\mathscr {C}_A$ from the server in the $(i+2){th}$ session, an attacker can follow these steps:

1. Eavesdrops on two consecutive sessions (sessions *i* and $i+1$) of the protocol involving $\mathscr {C}_A$, and extracts the secret parameters $\{R^{i+2}_{A};R^{i+2}_{B};C^{i+2}_{A};C^{i+2}_{B}\}$.
2. In the $(i+2){th}$ session, the attacker can impersonate $\mathscr {C}_A$ and update its CRP responses to $C^{i+3}_{A}=C^i_{A}\oplus R^i_{A}$ and $R^{i+3}_{A}=PUF^A(C^{i+3}_{A})$ during the update phase of the authentication process. The attacker can then send $<M^i_{SA},H^i_{SA}>$ to the server. It is enough for the attacker to assign a random value to $R^{i+3}_{A}$ at this stage.
3. The server will accept the received message and update $\mathscr {C}_A$’s record of the CRP to $(C^{i+3}_{A};R^{i+3}_{A})$.

However, there is only a probability of $2^{-n}$ that the random value assigned to $R^{i+3}_{A}$ by the attacker will match the value obtained from the PUF function $PUF^A(C^{i+3}_{A})$. Therefore, the success probability of the attack is $1-2^{-n}$ and the complexity of the attack is three consecutive sessions of the protocol.

## Security analysis of EV-PUF

In^16, TABLE XI]^, the designers claimed the security of EV-PUF against various attacks, including impersonation, privileged insider, password leakage, stolen smart card attacks, and satisfying forward secrecy. In this section, we evaluate the security of EV-PUF in an adversary model similar to that of the designers^16, Sec. III.B]^, i.e. Dolev–Yao model^31^ and the CK-adversary model^32^ to shed light on the security of the protocol from an independent third-party point of view. We acknowledge that launching impersonation attacks or conducting insider attacks can be challenging in a time-constrained environment, e.g. during the handover process. However, it is important to note that our evaluation of the EV-PUF protocol is based on the security claims made by its designers. As mentioned in the threat model, adversaries attempt to acquire sensitive information by launching passive or active attacks on communications transmitted through a public channel among communicative parties. Our proposed attacks against the EV-PUF protocol are based on the same assumption. We appreciate the practical difficulty of executing certain types of attacks within the stringent time constraints of the charging phase and the handover process for instance. Nevertheless, it is essential to thoroughly evaluate the security of any protocol against the potential threats specified in the designated threat models. By identifying vulnerabilities and proposing improvements, we aim to enhance the overall security and resilience of the EV-PUF protocol.

### The lack of forward secrecy

A protocol provides forward secrecy if compromising long-term secrets does not compromise the confidentiality of the data transferred in previous sessions, even if the adversary has eavesdropped on the entire communication in those sessions. In other words, if an adversary can compromise the long-term secret of a party after the completion of a session *j*, it should not be able to determine the session key used in a previous session *i*.

Forward secrecy is typically achieved by using ephemeral keys, which are generated for each session and are not stored after the session’s completion. If an adversary compromises a party’s long-term secret, it will not be able to derive the session key for any previous session because the session key was derived from an ephemeral key that has been discarded.

In general, forward secrecy is an important security property that provides protection against attacks that rely on the compromise of long-term secrets. By utilizing ephemeral keys, protocols can ensure that, even if an adversary gains access to long-term secrets, the confidentiality of previous sessions remains intact.

Following Section  “EV-PUF”, the shared key is computed as $SK=\mathscr {H}(RN_2\Vert RN_3\Vert R^1_i\Vert R^{2+}_i)$ and the transferred messages over the channel are $<B,D,RN_1,n^*_2,V_1>$, $<C_i,R^{1*}_i,n^*_3,V_2>$ and $<R^{*}_{i+1},X,V_3>$ where:

$$\begin{aligned} B = &\; {} PID_{EV}\oplus \mathscr {H}(ID_{RSU_i}\Vert n-1\Vert \mathscr {H}(SK_{RSU_i})) \\ D = &\; {} \mathscr {H}(PID_{EV}\Vert Q_S\Vert RN_1\Vert B) \\ n^*_2 = &\; {} RN_2\oplus k_i \\ V_1 = &\; {} \mathscr {H}(n^*_2\Vert k_i) \\ n^*_3 = &\; {} RN_3\oplus k_i \\ R^{1*}_i = &\; {} R^1_i\oplus k_i \\ V_2 = &\; {} \mathscr {H}(n^*_3\Vert k_i\Vert R^{1*}_i\Vert RN_2) \\ X = &\; {} R^{2+}_i\oplus k_i \\ R_{i+1} = &\; {} PUF(C_{i+1}) \\ R^{*}_{i+1} = &\; {} R_{i+1}\oplus k_i \\ V_3 = &\; {} \mathscr {H}(k_i\Vert R^{*}_{i+1}\Vert RN_3\Vert X) \end{aligned}$$

On the other hand, from “EV-PUF”, TSP stores $$<PID_{EV}, C_i, R_i, k_i,R_x)>$$ in a table entitled $$T_{CRP}$$ and $$k_i$$ is not updated. Therefore, it is reasonable to consider adversarial access to these data while evaluating the forward secrecy property of EV-PUF. Assuming that the adversary has $$k_i$$ and the transferred data over the public channel, it does the following computations:

$$\begin{aligned} RN_2 = &\; {} n^*_2\oplus k_i \\ RN_3 = &\; {} n^*_3\oplus k_i \\ R^{1}_i = &\; {} R^{1*}_i\oplus k_i \\ R^{2+}_i = &\; {} X\oplus k_i \\ \end{aligned}$$

which is enough to recover the session key $SK=\mathscr {H}(RN_2\Vert RN_3\Vert R^1_i\Vert R^{2+}_i)$. Hence, despite the designers’ claim, this protocol does not meet forward secrecy.

### Impersonation attack

The protocol provides security against impersonation attacks if the adversary cannot impersonate any protocol’s party toward another party with non-negligible probability. On the other hand, after mutual authentication of EV, it can request a charge, where following Section “EV-PUF”, RSU chooses a seed $S_i$ to compute $KDF_{SK}(S_i)=K_1\Vert K_2$ and $V_4=\mathscr {H}(K_1\Vert K_2\Vert N_ct)$ and send $<S_i,V_4>$ to EV. It also computes $Tag=\mathscr {H}(K_1\Vert K_2\Vert PID_{EV}\Vert ID_{RSU_i}\Vert N_{ct})$ and sends $<E_{Gpad}(Tag)>$ to the $1^{st}$ CP. Once the message has been received, EV recomputes $KDF_{SK}(S_i)=K_1\Vert K_2$ and $V_4=\mathscr {H}(K_1\Vert K_2\Vert N_ct)$ to verify the correctness of $V_4$. Then it calculates $Tag=\mathscr {H}(K_1\Vert K_2\Vert PID_{EV}\Vert ID_{RSU_i}\Vert N_{ct})$ and sends $<Tag>$ to the $1^{st}$ CP. The first CP compares the *Tag* received from EV and the encrypted one from RSU. However, an adversary can eavesdrop on the transferred messages toward CP and replay them at any time and receive a charge illegitimately.

### Privileged insider attack

A privileged insider adversary is assumed to have more capability compared to an inherent adversary. A common capability could be its access to the stored data in the memory of the transferred data through secure channels, for example during the registration phase of a protocol^37,38^. Following this fact, we evaluate the security of EV-PUF. It is clear *TSP* stores $$<PID_{EV}, C_i, R_i,k_i, R_x)>$$ in a table entitled $$T_{CRP}$$ and $$k_i$$ is not updated. Hence, similar to the attack process described in Section “The lack of forward secrecy” given $$k_i$$ and the messages transferred over the public channel, the adversary could recover the shared session key between EV and RSU.

Another type of insider could be malicious $EV_i$. Such adversary has access to $\mathscr {H}(SK_{RSU_i})$ and $ID_{RSU_i}$. It is worth noting that $ID_{RSU_i}$ could also be accessed from the public channel because it is transferred plainly from RSU to EV during handover from $RSU_j$ towards $RSU_i$ for instance. However, we need a malicious EV or other insiders for $\mathscr {H}(SK_{RSU_i})$. Given $\mathscr {H}(SK_{RSU_i})$ and $ID_{RSU_i}$ and also $<B,D,RN_1,n^*_2,V_1>$ which is sent to the RSU by $EV_j$ in Section “EV-PUF”, where $B=PID_{EV_j}\oplus \mathscr {H}(ID_{RSU_i}\Vert n-1\Vert \mathscr {H}(SK_{RSU_i}))$ the adversary can retrieve $PID_{EV_j}$. Hence, the adversary which has access to just a single malicious $EV_i$ is able to retrieve the $PID_{EV_j}$ of any target $EV_j$, once it participates in a login and authentication session with $RSU_i$. This violates the privacy of the location of electric vehicles.

### Pandemic attack

Let’s assume the adversary compromised a node, e.g. $EV_i$. In this case following Section “The lack of forward secrecy”, the adversary is able to access $ID_{RSU_i}$ and $\mathscr {H}(SK_{RSU_i})$ also retrieve $PID_{EV_j}$ for any ${EV_j}$ which communicates with $RSU_i$, from $B=PID_{EV_j}\oplus \mathscr {H}(ID_{RSU_i}\Vert n-1\Vert \mathscr {H}(SK_{RSU_i}))$. This information is enough to consider this protocol as a victim of the pandemic attack.

A consequence of this attack is to do RSU impersonation and connect the target $EV_j$ to a desired $RSU_j$, which could be a malicious one even. More precisely, following Section “EV-PUF”, the target EV, for which the adversary already extracted its $PID_{EV_j}$ through the pandemic attack, sends a handover query. In this point, the adversary decides the target RSU, e.g. $RSU_m$, and sends $<RN_4,ID_{RSU_m}>$ to EV and sends $<E_{PU_{RSU_m}}(PIDV_{EV_j},RN_4,ID_{RSU_i})>$ to $RSU_m$. Using its $T_{EV}$, the EV cross-checks the received $ID_{RSU_m}$ to compute $G=\mathscr {H}(PID_{EV_j}\Vert ID_{RSU_m}\Vert RN_4\Vert ID_{RSU_m})$ and send it to $RSU_m$ and also computes $SK_{T}=\mathscr {H}(PID_{EV_j}\Vert RN_4)$. On the other hand, $RSU_m$ decrypts the received message from $RSU_i$ and once again computes $G=\mathscr {H}(PID_{EV_j}\Vert ID_{RSU_i}\Vert RN_4\Vert ID_{RSU_m})$ to authenticate EV and compute $SK_{T}=\mathscr {H}(PID_{EV}\Vert RN_4)$. At the end of the process, the adversary impersonated $RSU_i$ successfully and could also access the shared $SK_{T}=\mathscr {H}(PID_{EV}\Vert RN_4)$.

## PUF-based mutual authentication protocol

In this section, we propose a PUF-based mutual authentication protocol for IoT systems with forward secrecy. Besides a PUF, we also use Ascon cipher suite^18^ to provide confidentiality and integrity of the transferred messages, including Ascon-128 as an authenticated cipher and Ascon-Hash as a cryptographic hash function. Ascon has been designed to be easy to implement, scalable, and resistant to timing and side-channel attacks. Ascon has been selected by NIST for future standardization of lightweight cryptography and is recommended for resource-constrained environments. For an authenticated cipher, if *K*, *N*, *A*, *P*, *C*, $\mathcal {T}$ are respectively key, nonce, associated data, plaintext, ciphertext, and the integrity check-value, then^6^:

$$\begin{aligned}E_K(N,A,P)=(C,\mathcal {T}).\\D_K(N,A,C,\mathcal {T})=(P,\bot )\end{aligned}$$

Where, $E_K(\cdot )$ denotes encryption and $D_K(\cdot )$ denotes decryption. $\bot$ is a null value, i.e. if the integrity check fails the cipher does not return any things.

### System setup

Each IoT device is equipped with a physically unclonable function $PUF(\cdot )$ a fuzzy extractor $(\text {Gen}(\cdot ), \text {Rep}(\cdot ,\cdot ))$ and the cipher suite Ascon ($\mathscr {H}(\cdot )$, $E_K(\cdot )$ and $D_K(\cdot )$). For a fuzzy extractor, given an input *w*, if $\text {Gen}(w)=(x,y)$ then $\text {Rep}(w',y)=x$ if $FHD(w,w')<\tau$, where *y* is helper string.

### Device registration

When a new IoT device is manufactured, it should be registered through a secure channel with a trusted entity, such as a server or gateway, that holds a database of registered devices as follows:

1. The device generates an identity $ID_x$ and sends it to the server.
2. The server generates a random challenge $C_x$ and sends it to the device.
3. The device computes $R_x=PUF(C_x)$, $(R'_X,Help_x)=\text {Gen} (R_x)$, a session identifier $IDS^{old}_x=IDS^{new}_x=\mathscr {H}(R'_x,ID_x)$ and sends $\{Help_x,R'_x\}$ to the server and stores $(ID_x, IDS^{old}_x,IDS^{new}_x)$.
4. the server recompute $IDS_x=\mathscr {H}(R'_x,ID_x)$ and stores $\{C_x, R'_x,ID_x,Help_x\}$, indexed by $IDS_x$, in a secure memory.

### Mutual authentication protocol

**Figure 1:**
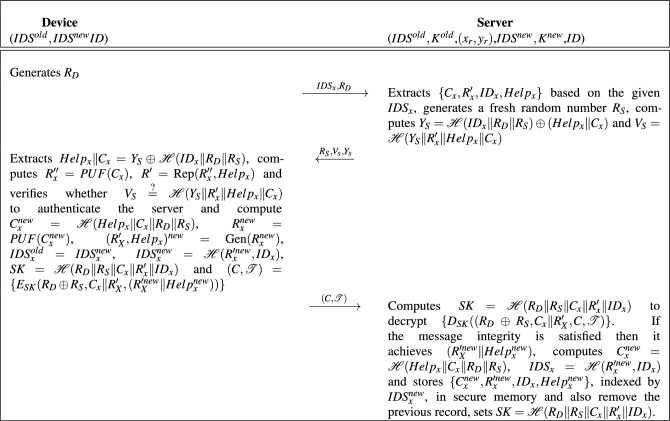
Mutual authentication phase of the proposed protocol.

The mutual authentication phase is as follows, also depicted in Fig. 1:

1. The device sends its session identifier $IDS_x$ along a fresh random number $R_D$ as $M_1=\{IDS_x,R_D\}$ to the server, over a public channel.
2. The server extracts $\{C_x, R'_x,ID_x,Help_x\}$ based on the given $IDS_x$. Then it generates a fresh random number $R_S$, computes $Y_S=\mathscr {H}(ID_x\Vert R_D\Vert R_S) \oplus (Help_x\Vert C_x)$ and $V_S=\mathscr {H}(Y_S\Vert R'_x\Vert Help_x\Vert C_x)$ and sends $M_2=\{R_S,V_s,Y_s\}$ to the device, over public channel.
3. The device extracts $Help_x\Vert C_x=Y_S\oplus \mathscr {H}(ID_x\Vert R_D\Vert R_S)$, computes $R''_x=PUF(C_x)$, $R'=\text {Rep} (R''_x,Help_x)$ and verifies whether $V_S\overset{?}{=}\mathscr {H}(Y_S\Vert R'_x\Vert Help_x\Vert C_x)$ to authenticate the server. If the server has been authenticated successfully, the device computes $C^{new}_x=\mathscr {H}(Help_x\Vert C_x\Vert R_D\Vert R_S)$, $R^{new}_x=PUF(C^{new}_x)$, $(R'_X,Help_x)^{new}=\text {Gen} (R^{new}_x)$, $IDS^{old}_x=IDS^{new}_x$, $IDS^{new}_x=\mathscr {H}(R'^{new}_x,ID_x)$ and $SK=\mathscr {H}(R_D\Vert R_S\Vert C_x\Vert R'_x\Vert ID_x)$ and sends $(C,\mathcal {T})=\{E_{SK}(R_D\oplus R_S, C_x\Vert R'_X, (R'^{new}_X\Vert Help^{new}_x))\}$ to the server as $M_3$.
4. The server computes $SK=\mathscr {H}(R_D\Vert R_S\Vert C_x\Vert R'_x\Vert ID_x)$ to decrypt $\{D_{SK}((R_D\oplus R_S, C_x\Vert R'_X, C,\mathcal {T})\}$. If the message integrity is satisfied then it achieves $(R'^{new}_X\Vert Help^{new}_x)$ as the decrypted plaintext. Next it computes $C^{new}_x=\mathscr {H}(Help_x\Vert C_x\Vert R_D\Vert R_S)$, $IDS_x=\mathscr {H}(R'^{new}_x,ID_x)$ and stores $\{C^{new}_x, R'^{new}_x,$ $ID_x,Help^{new}_x\}$, indexed by $IDS^{new}_x$, in a secure memory and also remove the previous record.
5. The session key is defined as $SK=\mathscr {H}(R_D\Vert R_S\Vert C_x\Vert R'_x\Vert ID_x)$.

It is worth noting that in Ascon’s computation, the nonce is derived as the XOR of $R_D$ and $R_S$, while the associated data is formed by concatenating $C_x$ and $R'_X$. Although this information could be transmitted over a channel, it is not necessary in this protocol because both entities already possess this information.

### Security and cost evaluation of the proposed protocol

In this section, prior to conducting a comparison with other protocols, we present an informal argument for the security of the proposed protocol against various attacks.

#### Replay attacks

The protocol incorporates session-dependent nonces, $R_D$ contributed by the device and $R_S$ contributed by the server, as part of the authentication process. Additionally, the identifier is updated for each new session, rendering previous authentication data invalid. These measures effectively prevent replay attacks by ensuring the freshness of the authentication process. The nonces are utilized to challenge the device/server, making it almost impossible to replay previously recorded responses and maintaining the integrity of the protocol.

#### Impersonation attacks

The protocol incorporates session-dependent nonces, $R_D$ contributed by the device and $R_S$ contributed by the server. Additionally, several other messages are transferred over public channels, including $Y_S$, $V_S$ and $(C, \mathcal {T})$, where:

$$\begin{aligned} Y_S = &\; {} \mathscr {H}(ID_x\Vert R_D\Vert R_S) \oplus (Help_x\Vert C_x) \\ V_S = &\; {} \mathscr {H}(Y_S\Vert R'_x\Vert Help_x\Vert C_x) \\ (C,\mathcal {T}) = &\; {} \{E_{SK}(R_D\oplus R_S, C_x\Vert R'_X, (R'^{new}_X\Vert Help^{new}_x))\} \\ SK = &\; {} \mathscr {H}(R_D\Vert R_S\Vert C_x\Vert R'_x\Vert ID_x)\\ \end{aligned}$$

These messages play a crucial role in preventing impersonation attacks. To impersonate the server, an adversary $\mathcal {A}$ would need to compute a valid $(Y_S, V_S)$ for a given $R_D$. However, this task is infeasible without at least the knowledge of $R'_x$. Importantly, $R'_x$ is never transmitted in plain text over the public channel, making it highly challenging for an adversary to acquire this critical information.

On the other hand, to impersonate the device, $\mathcal {A}$ would need to provide a valid $(C, \mathcal {T})$, which again is not feasible without at least the knowledge of $R'_x$. This safeguard ensures that unauthorized devices or servers cannot impersonate valid devices or servers, as the required information for constructing valid $(C, \mathcal {T})$ remains protected.

By relying on the secrecy of $R'_x$ and ensuring its non-disclosure over public channels, the protocol effectively prevents impersonation attacks and maintains the integrity and authenticity of the communication between devices and servers.

#### Session key compromise attack

The protocol does not directly address session key compromise attacks. However, each session uses a new set of ephemeral key pairs. In the event of a compromise of a session key, assuming that the adversary missed at least the data of a session after it, the impact is limited to that specific session, ensuring forward secrecy. Other sessions and their associated keys remain secure.

#### Secret key extraction

The session key, denoted as *SK*, is derived by applying the hash function $\mathscr {H}$ to the concatenation of $R_D$, $R_S$, $C_x$, $R'_x$, and $ID_x$, i.e., $SK = \mathscr {H}(R_D \Vert R_S \Vert C_x \Vert R'_x \Vert ID_x)$. Extracting the session key would necessitate knowledge of $(C_x \Vert R'_x \Vert ID_x)$, which is practically unattainable without possessing at least the values of $R'_x$ and $ID_x$.

Furthermore, it is crucial to emphasize that these values are always transmitted in an encrypted format during the communication process. This additional security measure reinforces protection against unauthorized access or extraction by adversaries. By ensuring the confidentiality of the transmitted data, the protocol significantly mitigates the risks associated with unauthorized key extraction or access to sensitive information.

#### Desynchronization attack

The proposed PUF-Based Mutual Authentication Protocol can also provide security against desynchronization attacks. A desynchronization attack aims to disrupt the synchronization between the device and the server, potentially leading to authentication failures or unauthorized access. Permanent authentication failure requires an unauthorized impersonation. More precisely, to launch such a desynchronization attack, an attacker would need to disrupt the synchronization between the device and the server by impersonating one of them. Given that the proposed protocol is secure against replay attacks and impersonation attacks the protocol does not suffer from this attack. In addition, in the protocol, the mutual authentication process involves the exchange of random numbers between the device and the server. These random numbers are crucial for establishing a secure session key and ensuring the freshness of the communication. In addition, if a desynchronization occurs due to the blocking of the last message, it can be synchronized again, because the device keeps the history of old *IDS*. However, the security of the protocol relies on the assumption that both parties faithfully execute the protocol steps. Any deviation or failure to correctly follow the protocol would likely result in authentication failure or termination of the session which is not the subject of this analysis

#### PUF-CRP extraction

The protocol leverages the security of the embedded PUF to generate unique responses. To enhance the reliability of these responses, a fuzzy extractor is employed. This mechanism significantly increases the difficulty for an attacker to extract the PUF-CRP (Challenge-Response Pair) and impersonate a device. Importantly, neither $C_x$ nor $R_x$ are transmitted in plain text during the protocol execution, and they are always masked to provide an additional layer of protection against potential attacks, including modeling attacks.

#### Man-in-the-middle attacks

The proposed PUF-Based Mutual Authentication Protocol provides security against Man-in-the-Middle (MitM) attacks. A Man-in-the-Middle attack occurs when an adversary intercepts and manipulates the communication between the device and the server, impersonating each party to establish a false sense of trust. Given that the proposed protocol is secure against impersonation attacks it does not suffer from MitM also. In addition, the protocol includes a challenge-response mechanism during the mutual authentication phase. The device and the server exchange random numbers and perform cryptographic operations based on these values. This process ensures that both parties can verify each other’s authenticity and integrity. By verifying the exchanged values, the device and the server can detect any tampering or modifications introduced by an attacker attempting a Man-in-the-Middle attack. Moreover, the protocol utilizes the Ascon cipher suite, specifically Ascon-128, for encryption and decryption. Ascon provides strong cryptographic primitives, including symmetric encryption and authentication, to protect the confidentiality and integrity of the communication. These cryptographic operations ensure that the exchanged messages cannot be tampered with or modified by an attacker without being detected by the recipient.

#### Privileged insider adversaries

The protocol assumes that the trusted entity is honest and does not leak any confidential information. As long as the trusted entity maintains the confidentiality of the device record, i.e. $\{C_x, R'_x,ID_x,Help_x\}$, privileged insider attacks are mitigated.

#### Pandemic attack

The security of the proposed PUF-Based Mutual Authentication Protocol against pandemic attacks is ensured by design. Since the transferred messages are the sole parameters specific to each device, compromising one device does not impact the security of other devices. This property ensures that the protocol remains secure even if an attacker gains unauthorized access to one device, preventing the spread of security breaches to other devices.

#### Traceability attack

The proposed protocol ensures the absence of traceability of devices or sessions, thereby enhancing privacy and security. Specifically, by excluding *IDS* and relying on session-dependent ephemeral keys $R_D$ and $R_S$, all transferred messages are either fresh or influenced by these session-specific keys. Additionally, *IDS* is updated after each successful session, further preventing the adversary from linking two successful sessions or identifying a participant device across independent successful sessions within the protocol.

However, it is important to note that if a device has not participated in a successful session, its *IDS* remains unaffected and can be traced. Furthermore, the device retains the old record of *IDS* to avoid desynchronization. Consequently, the adversary possesses the ability to trace the device after a single successful session, but not beyond that.

### Performance and security comparison

In Table 2, we present a security comparison of the proposed protocol with its predecessors, namely EV-PUF and PLAKE, based on the conducted security analysis in this study. It is clear the proposed protocol provides better security compared to those protocols.

To provide a comprehensive cost comparison of the proposed protocol with other related protocols beyond the scope of this paper, we present a comparison in Table 3. This comparison follows the approach outlined in^39^, where an Arduino UNO is used as the testbed. The table includes timings for the hash function ($T_h$), PUF invocation ($T_{PUF}$), two ECC scalar-multiplications ($T_{ECC}$), a double scalar-multiplication ($T_{2ECC}$), and symmetric encryption ($T_{Es}$). We assume $Ti_{ECC}\approx 21$ ms, $Ti_{2ECC}\approx 26$ ms, $Ti_{h}\approx 3$ ms for SHA-256, and $T_{Es}=3.7$ ms. The time for a PUF invocation ($Ti_{PUFn}$) is assumed to be equal to $Ti_{h}$. Following^40^, we approximate $Ti_{FHD.GEN}$ and $Ti_{FHD.REC}$ to be $10\times Ti_{PUF}$ and $30\times Ti_{PUF}$, respectively.

Based on the presented results, the computation and communication of the proposed protocol appear to be reasonable, e.g. it has the lowest communication overhead among the compared protocols. However, it is important to note that the reliability of the PUF response is not perfect. Therefore, it is necessary to incorporate auxiliary functions such as a fuzzy extractor to ensure a reliable output. On the other hand, some references may consider the PUF as ideal and reliable in their analyses. However, in practical implementations, the inclusion of auxiliary functions increases the overall expected time for these schemes. This is due to the additional processing required for the application of the mentioned auxiliary function.

**Table 2:** Security comparison of the improved protocol to EV-PUF and PLAKE, where **Imp.**, **S.D**, **Des.**, **Ins.**, **Pan.**,**F.S.** respectively denote the protocol vulnerability against impersonation attack, secret disclosure attack, desynchronization attack, privileged insider attack, pandemic attack, and the lack of forward secrecy. $^*$ the proposed protocol provides conditional forward secrecy..

Protocol	Imp.	S.D	Des.	Ins.	Pan.	F.S.
EV-PUF^16^	$\times$	$\checkmark$	$\checkmark$	$\times$	$\times$	$\times$
PLAKE^15^	$\times$	$\times$	$\times$	$\checkmark$	$\checkmark$	$\checkmark$
The proposed schemes	$\checkmark$	$\checkmark$	$\checkmark$	$\checkmark$	$\checkmark$	$\checkmark$ $^*$

**Table 3:** Cost comparison of the proposed protocol versus some related protocol .

Protocol	Computations	Time (ms)	Communications (bits)
^41^	$19\times T_{h}+4\times T_{Es}+8\times T_{ECC}$	240	2912
EV-PUF^16^	$16\times Ti_{h}+ 2\times Ti_{PUF}+Ti_{FHD.GEN} +Ti_{FHD.REC}$	174	2176
^42^	$5\times Ti_{h}+6\times Ti_{ECC}+ 2\times Ti_{2ECC}$	193	1632
^43^	$11\times Ti_{h}+6\times Ti_{ECC}+ 2\times Ti_{2ECC}$	211	1600
PLAKE^15^	$6\times Ti_{h}+ 2\times Ti_{PUF}$	24	1536
^44^	$10\times Ti_{h}+8\times Ti_{ECC}$	198	1440
EPSG^39^	$9\times Ti_{h}+ Ti_{PUF}+6\times Ti_{ECC}$	156	1408
^45^	$8\times Ti_{h}+6\times Ti_{ECC}+ 2\times Ti_{2ECC}$	202	1344
Proposed	$10\times Ti_{h}+ 2\times Ti_{Es}+ 2\times Ti_{PUF}+Ti_{FHD.GEN} +Ti_{FHD.REC}+$	161.4	1280

## Conclusion

In this paper, we analyzed two PUF-based security protocols, PLAKE, a mutual authentication protocol for IoT systems, and EV-PUF, an authentication protocol for dynamic charging systems of electric vehicles. Specifically, we show that PLAKE and EV-PUF are subject to a variety of attacks that can compromise the security of the systems they are designed to protect, including spoofing and key compromise attacks. It is worth noting that the proposed attack against PLAKE can extract the shared secret key with negligible cost and computation simply by eavesdropping on two consecutive sessions. The proposed attack against EV-PUF is also efficient. Furthermore, we show that compromising a single client with the EV-PUF protocol can compromise the security of the entire network, making it vulnerable to pandemic attacks.

This study highlights that incorporating a promising component into a protocol may not guarantee its security. Besides secure primitives, the message structure also requires careful design to minimize the adversary’s advantage. In the case of PLAKE, the freshness of the protocol is not equally dependent on the contributions of the entities involved. A previous study^5^ demonstrated that such protocol is vulnerable to impersonation attacks, which also applies to PLAKE. To mitigate such attacks, all protocol parties must contribute to the protocol’s randomness or utilize timestamps.

Through the cryptanalysis of PLAKE and EV-PUF protocols, the research reveals significant vulnerabilities that compromise the security of these authentication schemes and highlight the need for enhanced security protocols. As a step in this direction, the research presents an improved PUF-based authentication protocol that addresses the identified vulnerabilities in PLAKE and EV-PUF. The proposed protocol mitigates the risks of impersonation attacks, key leakage, and pandemic attacks, providing stronger security guarantees for IoT systems or electric vehicle charging systems.

Overall, the research contributes to the field of PUF-based authentication protocols by identifying vulnerabilities in existing schemes and proposing an improved protocol that enhances security and resilience against various attack vectors.

## Data Availability

The datasets used and/or analysed during the current study available from the corresponding author on reasonable request.
